# A Delayed Advantage: Multi-Session Training at Evening Hours Leads to Better Long-Term Retention of Motor Skill in the Elderly

**DOI:** 10.3389/fnagi.2019.00321

**Published:** 2019-11-22

**Authors:** Carmit Gal, Ella Gabitov, Rinatia Maaravi-Hesseg, Avi Karni, Maria Korman

**Affiliations:** ^1^The Edmond J. Safra Brain Research Center for the Study of Learning Disabilities, University of Haifa, Haifa, Israel; ^2^Laboratory for Human Brain and Learning, Sagol Department of Neurobiology, Faculty of Natural Sciences, University of Haifa, Haifa, Israel; ^3^McConnell Brain Imaging Center, Montreal Neurological Institute, McGill University, Montreal, QC, Canada

**Keywords:** elderly, motor skill, multi-session training, retention, morning, evening, chronotype, actigraphy

## Abstract

The acquisition and retention of motor skills is necessary for everyday functioning in the elderly and may be critical in the context of motor rehabilitation. Recent studies indicate that motor training closely followed by sleep may result in better engagement of procedural (“how to”) memory consolidation processes in the elderly. Nevertheless, elderly individuals are mostly morning oriented and a common practice is to time rehabilitation programs to morning hours. Here, we tested whether the time-of-day wherein training is afforded (morning, 8–10:30 a.m., or evening, 6–9 p.m.) affects the long-term outcome of a multi-session motor practice program (10 sessions across 3–4 weeks) in healthy elderly participants. Twenty-nine (15 women) older adults (60–75 years) practiced an explicitly instructed five-element key-press sequence by repeatedly generating the sequence “as fast and accurately as possible.” The groups did not differ in terms of sleep habits and quality (1-week long actigraphy); all were morning-oriented individuals. All participants gained robustly from the intervention, shortening sequence tapping duration and retaining the gains (> 90%) at 1-month post-intervention, irrespective of the time-of-day of training. However, retesting at 7-months post-intervention showed that the attrition of the training induced gains was more pronounced in the morning trained group compared to the evening group (76 and 56.5% loss in sequence tapping time; 7/14 and 3/14 participants showed a > 5% decline in accuracy relative to end of training, respectively). Altogether, the results show that morning-oriented older adults effectively acquired skill in the performance of a sequence of finger movements, in both morning and evening practice sessions. However, evening training leads to a significant advantage, over morning training, in the long-term retention of the skill. Evening training should be considered an appropriate time window for motor skill learning in older adults, even in individuals with morning chronotype. The results are in line with the notion that motor training preceding a sleep interval may be better consolidated into long-term memory in the elderly, and thus result in lower forgetting rates.

## Introduction

Motor functioning and, specifically, the ability of older adults to acquire new fine motor skills and generate effective long-term procedural (“how to”) memory are often reduced compared to young adults (Voelcker-Rehage, 2008). The search for interventions that can attenuate the age-related decrements in the performance of the existing repertoire of motor skills, as well as in the ability to master newly acquired skills is of importance (King et al., 2013). The decline in motor learning abilities was suggested to reflect a general decrease in neuroplasticity with aging (King et al., 2013), or stricter control (“gating”) of the brain’s plasticity mechanisms subserving procedural long-term memory (Korman et al., 2015), or both. The latter notion implies that in specific bio-behavioral conditions, devised to meet the age-related constraints on plasticity, the potential of older adults to master motor skills may be better expressed.

One critical constraining or “gating” aspect of skill learning in the elderly is related to the circadian correlates of biological aging—the changes in activity-rest rhythms toward morning chronotype and the decrease in sleep quality and duration (Duffy et al., 2015). Aging is characterized by a blunted circadian rhythmicity in the core body temperature, cortisol, and melatonin, suggesting that changes in sleep architecture may be linked to weakened circadian regulation (Hood and Amir, 2017). Due to the morning-oriented preferred activity times, early awakening hours, and high day-time fatigue (Goldman et al., 2008), it is generally accepted that the assessment of cognitive functioning may be confounded by a decrease in alertness in the late afternoon or evening in the elderly (Hood and Amir, 2017); thus, scheduling training to evening hours is considered sub-optimal. Indeed, morning type older adults, accounting for more than 60% of the elderly population (Roenneberg et al., 2007; Fischer et al., 2017), often show prominent deterioration in cognitive performance over the day (May et al., 2005; Veneman et al., 2013; Tsokanaki et al., 2016). Nevertheless, recent studies suggest that time-of-day effects may differ across cognitive domains (Schmidt et al., 2015); implicit memory retrieval may, in fact, be better at off-peak than at peak alertness hours in both young and elderly (May et al., 2005).

The acquisition of new motor skills has been extensively studied using different versions of the finger tapping task. This task is viewed as an ecologically relevant model for the acquisition of complex manual skills, from writing/typing to playing a musical instrument, that involves the explicitly guided concatenation of single movements into sequences (Friedman and Korman, 2012, 2016). Along the course of learning, sequential performance becomes progressively faster and smoother, without compromising, and sometimes, improving accuracy (Hikosaka et al., 2002; Sosnik et al., 2004). Mastering a novel motor sequence is a multi-session process, whereby each training session elicits both immediate (online) and delayed (offline) changes in movement speed and accuracy (Karni and Sagi, 1993; Karni et al., 1995, 1998; Korman et al., 2003). An important outcome of multi-session training in young adults is that the gains are both sequence and effector specific, and thus are only partially generalizable to the performance of new sequences (Karni et al., 1995; Korman et al., 2003).

Although there are age-related declines in baseline motor performance, the ability to learn within-session is well preserved in the elderly (Durkin et al., 1995; Howard et al., 2004; Yan et al., 2010; Ehsani et al., 2015; Korman et al., 2015) [a possible exception may be learning under conditions of high task complexity (Rieckmann and Bäckman, 2009)]. However, offline learning, expressed as delayed between-session gains in performance, is consistently reported to be impaired in older adults (Brown et al., 2009; King et al., 2013; Korman et al., 2015). These delayed gains are considered to be a behavioral hallmark of memory consolidation processes and are time-dependent and, specifically in relation to movement sequence learning, time-in-sleep dependent (Karni et al., 1998; Korman et al., 2003). Thus, the relative deficits in the generation of delayed, consolidation-phase, gains following a single training session scheduled to the morning or day hours (Korman et al., 2015) may accumulate over multi-session practice and manifest as an overall slower rate of learning in older adults (Spencer et al., 2007; Wilson et al., 2012).

In young adults, both the magnitude of the delayed gains in explicitly instructed motor sequence practice and the time-course of the evolution of these gains are dependent on post-training sleep, either night-time or day-time, or both (Walker et al., 2003; Korman et al., 2007; Doyon et al., 2009; Walker and Stickgold, 2010). The role of sleep is conceptualized both as protecting from behavioral interference by subsequent motor experience and as promoting memory stabilization and enhancement. Because changes in sleep, often negative, occur in advanced age (Phillips and Ancoli-Israel, 2001; Huang et al., 2002), these changes were linked to the decline in cognitive functioning in the elderly (Mander et al., 2017; Dzierzewski et al., 2018) and, specifically, to the decrease in motor learning abilities (King et al., 2013). A large number of studies indicate that sleep-dependent procedural memory consolidation phase gains in performance are particularly weakened in older adults (Spencer et al., 2007; Wilson et al., 2012; Albouy et al., 2013; King et al., 2013; Terpening et al., 2013; Backhaus et al., 2016). Nevertheless, when sleep is allowed immediately after an evening training session (Mantua et al., 2016) or napping is afforded after training at late morning hours (Korman et al., 2015), healthy elderly were shown to generate significant overnight delayed consolidation phase gains in performance. It may be the case that evening training with proximity to a sleep period may promote better memory consolidation and minimize unspecific interference from everyday motor activity (Korman et al., 2015).

A critical aspect in evaluating the effectiveness and utility of any training intervention beyond the robustness of the acquired gains in performance is the durability of the skill. In the serial reaction time task single-session learning, both general (faster reaction times) and sequence-specific knowledge were retained, though not fully, over a 1-year period by older adults (Romano et al., 2010). Robust retention was reported in mirror-tracing skills in healthy older adults who practiced for 3 separate days and were retested 5 years later (Rodrigue et al., 2005). However, other evidence suggests that aging is related to faster forgetting rates (Malone et al., 2016; Griffin et al., 2017).

Given that, on the one hand, training in the morning hours may meet the circadian preference of older adults, but, on the other hand, that morning training may be less beneficial to the mastering of motor skills as a long time interval separates the practice experience from the interval of sleep at night; a direct comparison of the effects of affording a program of motor training at peak, and off-peak times, in the elderly, is warranted from both theoretical and practical perspectives. Here, using the well-established paradigm of the finger tapping sequence learning (FTSL) task (Karni et al., 1995, 1998), we investigated whether the time-of-day wherein training is afforded (morning or evening) is a significant factor in motor skill acquisition, the ability to generalize the gains in performance, and importantly, the retention of the skill, in healthy elderly participants. The effects of an extensive, 10-session training intervention were assessed in terms of sequence completion time and between key-press transition times [model task of motor sequence learning (Friedman and Korman, 2012)]. Accuracy levels, as markers of a possible speed-accuracy trade-off, were assessed in terms of the percentage of correct transitions from the total number of transitions (= 59 per block).

## Materials and Methods

### Participants

The study was approved by the University of Haifa Human Experimentation Ethics Committee. All participants gave written informed consent of participation in the study after being provided with explanations of its purpose. Twenty-nine active, community-dwelling elderly participants (60–75 years old; mean age = 67.3 ± 4.23, 16 women) took part in the current study. In the evening group: 9/15 = 60% were women and in the morning group 7/14 = 50% were women. The group size of the current exploratory study was based on the effect sizes of the offline memory consolidation effects in terms of sequence duration, observed in the same task in Korman et al. (2015) (with healthy elderly participants): the group that had a nap immediately after training improved by 17%, whereas the group that did not nap improved by 4%, when re-tested 24 after the training. The Eve group was expected to show better overnight memory consolidation, with similar differences in performance improvement following training afforded during morning hours compared to evening hours, due to proximity to the sleep interval of the latter. Based on a standard deviation in each group of 11.5%, and a power of 0.95, 15 subjects in each group were required, based on a mixed-design ANOVA. No previous data were available to perform power analysis for the effects of multi-session training; the differences in the long-term representation of the skill after a single training session were expected to accumulate over the course of multi-session training.

The participants were recruited through public advertisements and a “snow-ball” approach from a single kibbutz in northern Israel, if meeting the basic requirements of good health and right-handedness. All participants were retired from their permanent work, however, engaged in variable jobs in the kibbutz, for at least 4 hours a day, during the study period. Individuals with neurological, psychiatric, or musculoskeletal system disorders, unstable cardiovascular status, users of psychotropic medication, those who have been diagnosed with diabetes, sleep disorders/insomnia, or impaired thyroid function, as well as overweight individuals were excluded. Since there is no BMI cut-off score that is universally accepted in older adults (Yan et al., 2004), the inclusion criterion in the current study was BMI < 27 (upper limit of normal BMI in young adults is 24.9). People designating themselves as heavy smokers and heavy alcohol and caffeine consumers (> 3 drinks per day on average); professional musicians or/and professional typists; as well as shift-workers, were excluded from the experiment. Participants reporting frequent, habitual, day-time napping were also excluded.

All participants were first evaluated using a structured telephone interview. Inclusion criteria were thoroughly verified at the first meeting, using standard questionnaires for general health and sleep-activity habits. All participants were right-handed according to the Edinburgh handedness inventory (scored > 50 points) (Oldfield, 1971). Emotional health was evaluated using the Beck’s anxiety and depression inventory (Steer et al., 1986). Given the recognized effects of sleep on learning, the sleep–wake parameters of participants were assessed using the Pittsburg Sleep Quality Index (PSQI) (Buysse et al., 1989), Daytime Sleepiness Questionnaire (Epworth: Johns, 1991), and the Morningness–Eveningness Questionnaire (MEQ) for assessment of the circadian type (Horne and Ostberg, 1976).

Participants meeting the inclusion criteria were randomly assigned to one of the study groups, using the block randomization method: to be trained either in the morning (8–10:30 a.m.), or in the evening (6–9 p.m.) hours. No differences were found between the groups in terms of age and level of education, and in scores of the screening questionnaires (Table 1).

**TABLE 1:** Mean scores for demographic data and screening questionnaires, by group.

	**Variable**	**Morning *N* = 14**	**Evening *N* = 15**	**Interpretation**
		**Mean + *SD***	**Mean + *SD***	
	Age	62.7 ± 5.86	63.5 ± 5.09	
	Education (years)	11.64 ± 1.33	11.14 ± 1.02	
	Handedness score	89.19 ± 18.53	100	
Emotional health	Beck anxiety inventory	2.53 ± 4.37	3.64 ± 3.91	> 9 minimal anxiety
	Beck depression inventory	2.66 ± 4.12	2.94 ± 3.11	> 9 minimal depression
Sleep and wake	Epworth	5.92 ± 4.92	5.0 ± 3.08	> 10 excessive sleepiness
	PSQI	4.691.88	5.3 ± 2.32	> 5 sleep disturbances
	MEQ	60 ± 8.7	63.15 ± 5.33	> = 59 morning type

### The Setup and the FTSL Task

**FIGURE 1:**
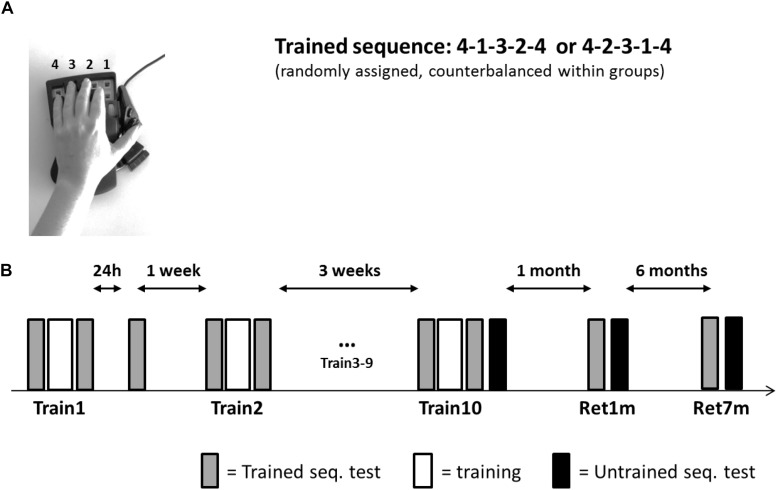
Setup, task, and training protocol. **(A)** The Finger Tapping Sequence Learning (FTSL) task. **(B)** The training protocol. One of the two sequences (4-1-3-2-4 or 4-2-3-1-4) was used as the trained sequence; its mirror reversed counterpart sequence was used in transfer tests (untrained sequence). Participants in both groups (evening and morning) were initially trained in a single training session (Train1), retested at 24 h post-training, and a week later continued in the multi-session training protocol—nine additional training sessions (Train2–Train10) spaced 2–3 days apart. Altogether, the training period spanned 3–4 weeks. Performance of the untrained (transfer) sequence was tested immediately after the 10th session (Train10) and the trained and untrained sequences were retested at 1 month (Ret1m) and 7 months (Ret7m) after the Train10 session. Each session included 14 blocks; gray bars—the four initial and the final four blocks of the session were used as tests of the trained sequence; white bars—six blocks of training; black bars—four test blocks on the untrained sequence.

All participants were trained and tested in their private home, by the same experimenter. Participants were seated in a quiet room that included a table and a chair; this location was used throughout the study. A 17-in screen laptop was positioned about 50 cm from the participant and the response box. The left hand of the participant was placed on the four numeric keys, arranged in an ergonomic position, of a response box (Expert gaming Keypad-Razer Nostromo), with key-to-number assignment from right to left; the little finger designated 4 (Figure 1A).

The task practiced in the current study was a computerized version of the finger opposition sequence learning task, initially developed by Karni et al. (1995) (Figure 1A) implemented as the FTSL task; i.e., with key presses substituting the opposition movements. All participants had no prior experience with the task. The task consisted of repeating (tapping), as quickly and accurately as possible, a sequence of five finger movements using the left, non-dominant hand. Two mirror-reversed sequences of equal length and complexity (41234 or 42314, Figure 1A) were used; each participant was randomly assigned one sequence for training (trained sequence) and the second sequence served for the transfer tests (untrained sequence).

### Training Protocol and Performance Assessments

The experiment involved 10 training (intervention) sessions and two long-term retention tests (Figure 1B). Following the first training session, a 24-h performance re-test was performed (data not reported here). A week later, sessions 2–10 were afforded, on separate days spaced 2–3 days apart; thus, the training phase spanned 3–4 weeks in total. Retention was tested at 1 and 7 months after the completion of the 10th session of the intervention.

The training times were the same as the testing times in both groups (± 30 mins) throughout the study: participants of the morning group were trained at 8–10:30 a.m. and the participants of the evening group were trained at 6:30–9:00 p.m. Each training session lasted approximately 30 min. Tests for the ability to transfer the gains acquired in practice to a novel sequence, the performance of the untrained, mirror-reversed sequence, were performed at the end of the 10th session (Train10) and at the end of the 1-month and 7-months’ retention tests (Ret1m and Ret7m, respectively). Performance of the trained sequence was always tested first.

Full explicit knowledge and a demonstration of the required movement sequence were provided prior to each training session by verbal instruction and a presentation, on the computer screen, of the numbered keys comprising the to be trained or tested sequence, by the experimenter. The participants interacted with the same experimenter throughout the study. The training session began only after three consecutive, correct iterations of the target sequence were executed by the participant, indicating that the participant understood the required sequence.

All training sessions were identical in structure. Each training session consisted of 14 blocks, each block comprised of 12 repetitions of the assigned sequence (i.e., each block consisted of a total of 60 key presses). A 30-s rest period was afforded between consecutive blocks. The average of the four initial and four final blocks were used in the analysis as the measures of performance at the beginning (pre-test) and end (post-test) of each training session, respectively (Doyon et al., 2009). Each of the retention tests, at 1 and 7 months’ post-intervention, also included four consecutive blocks of the performance of the trained sequence; four consecutive blocks were also used to assess performance in the transfer condition, the performance of the untrained sequence. Participants were instructed not to practice the experimental task between sessions. All participants confirmed at the beginning of each meeting they followed this instruction.

Before the beginning of each block of trials, the participants were instructed/reminded to continuously tap the sequence “as fast and accurately as possible,” using their left non-dominant hand; starting immediately after the onset of the “go” signal on screen (“green cross”). Participants were then instructed that at the end of each block, a “red cross”(“stop” cue) will appear, as the cue to stop tapping the sequence. The screen background remained black throughout the session. No feedback on performance was provided online or offline. Participants were instructed that occasional errors should not be corrected, and were required to continue the task without a pause even in the case of an error.

One day prior to the first session of training, each participant was asked to wear an actigraph (ActiGraph wGT3X, ActiGraph, LLC) in order to record the participant’s sleep-activity cycles. The participants were instructed to wear the actigraph on their non-dominant wrist (left), continuously for a 7-day period, with the exception of time spent during water activities (shower, etc.). In addition, participants were asked to keep a sleep diary with entries for each day of the week they wore the actigraph. At the conclusion of the 7 days’ period, actigraph devices were collected and the data were retrieved using Actilife6 software. Participants were instructed to maintain their usual sleep habits and avoid day-time napping throughout the whole experiment.

### Data Extraction and Statistical Analyses

The timing and corresponding number of each key-press were recorded. The motor sequence performance measures were derived from the time differences between two consecutive key presses within each correctly performed and completed sequence, as well as between sequences (the final key-press of a sequence and the first key-press of the next sequence) in each task block using a custom MATLAB script (The Mathworks, Inc., Natick, MA, United States, version:2007). The pairs of movements (transitions) for analysis were four *within-sequence transitions*—transition from finger 4 to 1, 1 to 3, 3 to 2, and 2 to 4 for participants training on sequence 4-1-3-2-4 or the corresponding four transitions for sequence 4-2-3-1-4 in participants assigned the latter sequence. In addition, *between-sequence transition* times, the transition time from end of one sequence (finger 4) to the first movement of the next iteration (finger 4), was computed for each block. The main behavioral measures of performance were: (*i*) speed, calculated as the mean correct sequence duration – sum of within-sequence transitions in a given trial. The sequence duration analysis included only the correctly performed and completed sequences during each block; (*ii*) accuracy, calculated as the percentage correct transitions from the total number of transitions (= 59 per block).

Data were analyzed using the Statistical Package for the Social Sciences (SPSS Statistics for Windows, Version 24; IBM Corp., Armonk, NY, United States). A Kolmogorov–Smirnov test for normality was used to test the distribution of the data on the primary outcome measures. The magnitude of learning the trained sequence was assessed using mixed repeated measures general linear models (GLMs) [with two groups (morning/evening) × 10 sessions (Train1–Train10) × two time-points for each session (pre-test, post-test)], carried out separately for each performance measure. Retention of skill in the execution of the trained sequence across the intervals of 1-month and 7-months post-intervention compared to the last training was assessed using repeated measures GLMs (Rm-GLMs) [two groups (morning/evening) × three time-points (post-test of Train10, Ret1m, Ret7m)], carried out separately for each performance measure. To test for the degree of specificity of the acquired motor sequence knowledge, performance of the trained sequence was compared to the performance of a novel, untrained, sequence using paired *t*-tests at Train10, Ret1m, and Ret7m. Because the error percentage (accuracy) was not normally distributed, the non-parametric Chi-square test was used for assessing changes in accuracy.

The raw activity scores from the actigraphy were translated to sleep–wake scores based on standard software (scoring algorithm) (Actilife6). Mean total sleep time (TST), sleep efficiency, sleep latency, and wake time after sleep onset (WASO), number of awakening, and average of awakening duration. To assess possible difference in sleep quality and duration between the intervention groups, the means of the groups for each sleep parameter were compared using independent sample uncorrected *t*-tests and Mann–Whitney test for the non-parametric sleep parameters. The significance level was at *p* ≤ 0.05.

## Results

### Effects of Multi-Session Practice Across the Intervention Interval

**FIGURE 2:**
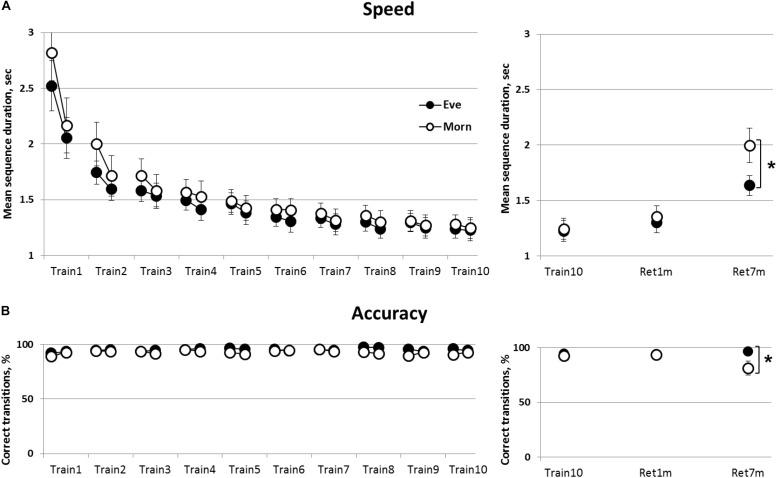
The time-course of learning and retention of a key-press sequence; evening (Eve) and morning (Morn) trained groups. Performance at the initial and the final practice blocks at each of the 10 practice sessions is shown (averages of first four and last four performance blocks, respectively) (left panels). **(A)** Speed—mean duration for correct sequences. **(B)** Accuracy—percent of correct transitions from the total number of key transitions (= 59 per block). The right panels show retention of the trained sequence at 1 (Ret1m) and 7 months (Ret7m) compared to the end of training (Train10). Open circles—morning group, filled circles—evening group. Bars—standard error of the mean (SEM), ^∗^—significant difference between the performance levels of the Eve and Morn groups, *p* ≤ 0.05.

Overall, both groups showed significant gains in mean correct sequence duration with no loss in accuracy (no speed-accuracy trade-off) (Figure 2A). Mixed Rm-GLM analyses comparing the performance across the 10 training sessions (time-point, between-sessions) for the initial and final four blocks of each session (within-session) in the two groups (Eve and Morn, between-subjects factor) showed that overall, there was a significant decrease in mean sequence duration, time-points [*F*(9,243) = 74.46, *p* < 0.001, η^2^ = 0.73]. There was, however, no significant main effect of group [*F*(9,243) = 0.65, *p* = 0.75, η^2^ = 0.024] and also no significant group × time-points interaction [*F*(1,27) = 0.58, *p* = 0.45, η = 0.021] (Figure 2A).

There was also a significant within-session effect [*F*(1,27) = 30.56, *p* < 0.001, η^2^ = 0.53] and a significant time-point × within-session effect [*F*(9, 243) = 23.40, *p* < 0.001, η^2^ = 0.46]; indicating that gains in performance speed (reduction of sequence duration) occurred within the initial sessions (sessions 1–3, 4; Train1–3,4 in Figure 2A).

However, there was no significant group × within-session or group × time-point × within-session interaction [*F*(1,27) = 0.58, *p* = 0.45, η^2^ = 0.021, *F*(9,243) = 1.33, *p* = 0.22, η^2^ = 0.05, respectively] indicating that the time-course of learning, within-sessions and across sessions, was independent of the time-of-day wherein the training sessions were afforded.

Accuracy throughout the training programs was high; with the number of correct sequences > 90% throughout the intervention sessions in both groups (Figure 2B). Non-parametric repeated measures Friedman tests showed that there was a trend toward a (small) increase in accuracy in the Eve trained group [χ2(19) = 28.65, *p* = 0.07] and no significant change in the accuracy of performance in the Morn group [χ2(19) = 18.07, *p* = 0.52] (Figure 2B). The gains in speed, therefore, evolved with no costs of accuracy, i.e., there was no speed-accuracy tradeoff.

The results, therefore, indicate that training at morning hours did not confer an advantage; both training protocols were effective in inducing learning across the 10 training sessions, without apparent differences in the magnitude of the learning gains or the time-course of within or between sessions acquisition of skill.

### Long-Term Maintenance of Skill

The retention of the gains in the performance of the practiced key-tapping sequence was tested at 1 month post-training and again by 7 months post-training (Figure 2, right panels). To assess retention, performance was compared across three time-points: the final training session, 1-month post-training and 7 months post-training (Train10, Ret1m, and Ret7m, respectively) (Figure 2). There was a significant deterioration in speed with sequence duration increasing significantly [*F*(2,52) = 27.57, *p* < 0.001, η^2^ = 0.52]. Nevertheless, although there was no significant group effect [*F*(1,26) = 1.2, *p* = 0.29, η^2^ = 0.04], there was a trend for a time-point × group interaction [*F*(2,52) = 2.97, *p* = 0.06, η^2^ = 0.10]. This trend reflected a greater deterioration in sequence duration from Train10 to Ret7m in the Morn group (from 1.28 to 1.99 s) than in the Eve group (from 1.25 to 1.63 s) (Figure 2A, upper right panel).

Participants practicing in the morning hours, the Morn group, tended also to show a deterioration in accuracy across the 7 months’ retention interval (Figure 2, lower right panel). Accuracy, in the Morn group, was reduced from 92 to 81% on average, but no losses were apparent in the Eve group (from 94 to 96%). A non-parametric χ2 (chi square) test was used to assess the proportion of participants in the two groups who experienced a loss of accuracy across the 7 months’ retention interval. To this end, we designated the participants, in the two groups, that experienced losses in accuracy on the order of 5% or more [comparing the last training session (Train10) to the retention test 7 months’ post-training]. The results showed that, in the Morn group, 7/14 participants showed a loss in accuracy; a proportion not statistically different from a 50:50 chance of either gaining and retaining or showing losses in accuracy [χ2(1) = 0.00, *p* = 1.0]. The proportion of participants showing such losses in accuracy during the retention interval, in the Eve group, was only 3/14; a proportion significantly different from a 50:50 chance of either improving or retaining the gains to showing losses [χ2(1) = 4.57, *p* < 0.05]. A direct comparison of the proportion of participants showing losses in accuracy (above 5%) over the 7 months’ retention interval showed a significant advantage for the Eve group [χ2(1) = 4.57, *p* < 0.05].

The between-group differences, in sequence duration and accuracy, at each time-point assessed using *post hoc t*-tests and Mann–Whitney *U*-tests comparisons are shown in Table 2.

**TABLE 2:** Between-group comparisons of the mean sequence duration and accuracy at three time-points: Train10, Ret1m, and Ret7m (group mean ± SD).

	**Train10**	**Ret1m**	**Ret7m**
Sequence duration, seconds	Eve: 1.22 ± 0.36,	Eve: 1.30 ± 0.36,	Eve: 1.63 ± 0.35,
	Morn:1.24 ± 0.35	Morn: 1.36 ± 0.36	Morn: 1.99 ± 0.58
	*t*(27) = −0.15, *p* = 0.884,	*t*(27) = −0.39, *p* = 0.7,	*t*(26) = −1.98, *p* = 0.05,
	*d*′ = 0.06	*d*′ = 0.17	*d*′ = 0.75
Accuracy, %	Eve: 94.43 ± 10.98,	Eve: 93.95 ± 14.35,	Eve: 96.4 ± 3.08,
	Morn: 92.70 ± 9.55	Morn: 93.79 ± 7.63	Morn: 81.3 ± 24.05
	*U*(29) = 95.5, *p* = 0.66, *d*′ = 0.17	*U*(29) = 79.5, *p* = 0.25, *d*′ = 0.01	*U*(28) = 55.0, *p* = 0.049, *d*′ = 0.88

Altogether, the results showed that by 7 months after the end of the intervention, there was a substantial forgetting of the skill, reflected in both sequence duration and accuracy, in the Morn group; however, the losses in speed were somewhat smaller in the Eve trained group, while accuracy was well maintained across the 7 months’ interval.

### Transfer

**FIGURE 3:**
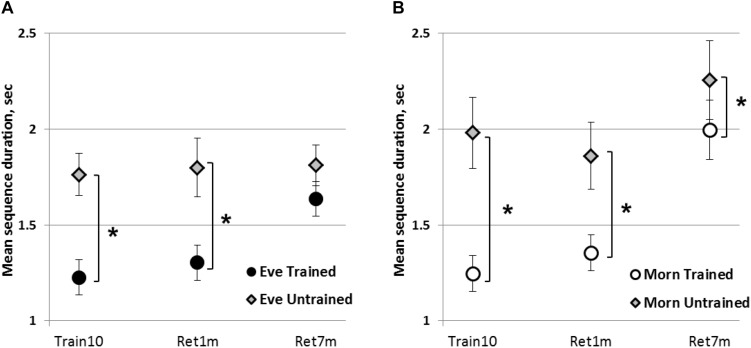
Generalizability of the acquired knowledge to the performance of the untrained sequence. **(A)** Eve group. **(B)** Morn group. Each data point represents the averaged performance of four test blocks in terms of mean duration for correct sequences. Train10—end of the last training session; Ret1m—1 month after the termination of training; Ret7m—7 months after the termination of training; error bars—SEM, ^∗^—significant difference between the performance levels of the trained and the untrained sequence, *p* ≤ 0.05.

To test the generalizability of the acquired knowledge, we compared the performance of the trained sequence to the performance of a newly introduced, untrained sequence, at the end of the intervention (Train10). The mean duration of completing a correct trained (Lt_T) and an untrained sequence (Lt_U) composed of the same component movements ordered in reverse to their order in the Lt_T sequence are presented in Figures 3A,B. There was a significant advantage for the Lt_T in both groups (Eve and Morn) in terms of mean sequence duration [*F*(1,27) = 62.80, *p* < 0.001, η^2^ = 0.7], with no significant sequence × group interaction [*F*(1,27) = 1.26, *p* = 0.272, η^2^ = 0.04] or group [*F*(1,27) = 0.75, *p* = 0.4, η^2^ = 0.03] effect. For accuracy, a non-parametric Friedman test of differences showed that in both groups, there was a significant difference in favor of the trained sequence [χ2(1) = 4.57, *p* < 0.05, χ2(1) = 7.14, *p* < 0.005; Eve and Morn, respectively]. Thus, in both groups, full transfer (generalization) of the acquired gains to the untrained condition did not occur.

We directly compared sequence duration for the trained sequence to the untrained sequence at 1 and 7 months’ post-training in the two groups, with time-point and sequence as within-subject’s effects. A mixed Rm-GLM analysis showed that there was a significant time-point effect [*F*(1,26) = 10.09, *p* < 0.005, η^2^ = 0.28] with no significant group × time-point interaction [*F*(1,26) = 2.28, *p* = 0.14, η^2^ = 0.08] or group effect [*F*(1,26) = 2.06, *p* = 0.16, η^2^ = 0.07]. There was a significant sequence effect [*F*(1,26) = 35.37, *p* < 0.001, η^2^ = 0.58] but no group × sequence interaction [*F*(1,26) = 0.04, *p* = 0.85, η^2^ = 0.01].

A non-parametric comparison for accuracy showed that there was no significant difference between the two sequences in the Eve group [χ2(1) = 1.33, *p* = 0.25] or in the Morn group [χ2(1) = 3.77, *p* = 0.12] at Train10. This was also the case at 7 months [χ2(1) = 0.0, *p* = 1.0; χ2(1) = 0.29, *p* = 0.59, Eve and Morn groups, respectively]. Thus, overall there was no significant difference between the two group in terms of transfer abilities.

### Sleep and Chronotype

Independent samples Mann–Whitney *U*-test showed no significant differences between the participants of the Morn and Eve groups in respect to chronotype as assessed by MEQ scores [*U*(28) = 73.0, *p* = 0.25, *d*′ = 1.14]. Parsing of the continuous MEQ scores into chronotype categories (Horne and Ostberg, 1976; Caci et al., 2009) showed that the participants of the study were mostly moderately morning chronotypes (Table 3).

**TABLE 3:** Chronotypes based on the MEQ scores in the two groups (Eve and Morn).

**Chronotype**	**Morn group**	**Eve group**
MEQ mean ±SD	60 ±8.7	63.15 ±5.33
Definitely morning type	1	1
Moderately morning type	11	14
Intermediate type	2	–
Moderately evening type	–	–
Definitely evening type	–	–

*The MEQ scores were parsed into categorical chronotypes using standard cut-off criteria (Horne and Ostberg, 1976; Caci et al., 2009).*

In the PSQI questionnaires, both the Eve (mean of 5.3 ± 2.32) and Morn (4.69 ± 1.88) groups’ scores were within the normal range (a score > 5 indicates poor sleep quality). Independent samples Mann–Whitney *U*-tests showed no significant differences between the two groups [*U*(26) = 73.0, *p* = 0.55, *d*′ = 0.29]. In the ESS, assessing the level of daytime sleepiness, there were also no significant differences between the two groups with no or only moderate levels of day time sleepiness (5 ± 3.08, 5.92 ± 4.92; the Eve and the Morn groups, respectively). The actigraphy-based sleep measures of the two groups were compared by averaging the sleep measures across seven nights, starting from the night of the first training session. The average scores of actigraphy-derived sleep parameters, including sleep efficiency, sleep onset latency, TST, total time in bed (TTB), wake after sleep onset (WASO), as well as the number of awakenings (Nu_awake) and average awakening time are shown in Table 4. There were no significant differences between the two groups (Eve, Morn) in any of these sleep parameters, suggesting that overall, the differences in the performance measures of the two groups may not be ascribed to differences in sleep habits, quality, and duration.

**TABLE 4:** Actigraphy parameters of the two groups (Morn, Eve), averaged across 7 days of recording during the first week of the training intervention.

**Sleep parameter**	**Morn group**	**Eve group**	**Significance**
Latency	5.29 ± 4.93	10.82 ± 11.42	*T*-test, ns
Efficiency	82.77 ± 7.63	82.96 ± 6.39	*U*-test, ns
TTB	409.91 ± 43.53	428.52 ± 51.71	*U*-test ns
TST	339.89 ± 58.83	353.43 ± 53.87	*T*-test, ns
WASO	64.63 ± 28.44	64.23 ± 24.38	*U*-test, ns
Nu_awake	14.86 ± 2.73	12.56 ± 31.86	*U*-test, ns
Avg_awake	4.37 ± 1.71	5.23 ± 1.72	*T*-test, ns

*A two-tailed independent samples *t*-test or a Mann–Whitney *U*-test was used to compare between the groups; ns, not significant; *p* > 0.05.*

## Discussion

In the current study, we tested whether the time-of-day wherein training was afforded (morning or evening groups) during a multi-session motor training program affects the acquisition and mastering of a movement sequence or the long-term outcome of the training program in healthy morning-oriented elderly participants. The results of the current study show that the multi-session intervention program, a total of 10 training sessions, resulted in robust gains in performance (mean sequence tapping duration and accuracy) irrespective of whether participants engaged in training on the task at morning or at evening hours. Thus, although evening hours are considered a non-preferred, “off peak,” circadian phase in most elderly, and in participants who were moderate to definite morning chronotype, the evening trained group was able to acquire experience-dependent gains in performance across the intervention period as robustly as their peers training at morning hours. Moreover, at 1 month after the end of intervention, both groups retained more than 90% of the gains attained by the end of the multi-session training program; but when re-tested at 7 months post-training, the Eve trained group was found to have a clear mnemonic advantage, showing significantly less attritions of the gains attained in training compared to the Morn trained group. Importantly, the mnemonic differences between the groups, in the current study, cannot be ascribed to differences in sleep parameters; no differences were found in the sleep parameters, subjective or objective, of the two experimental groups. The sleep assessments showed that all participants were moderately morning chronotype, and had age-adequate sleep.

Altogether, the current results show that a motor skill was effectively acquired by morning-oriented older adults; irrespective of whether training was afforded at morning or at evening. However, the current results also show that successful acquisition of skill across a multi-session training program may not suffice to secure the long-term retention of the skill; there was a significant deterioration in the performance of the task after an interval of a few months (a time interval wherein the task was not executed). Nevertheless, the time-of-day in which the training was afforded was a critical factor in determining retention; retention was better in the evening group compared to the morning group in terms of retaining the gains in speed and accuracy attained in the performance of the task during the practice program.

Multi-session training was found beneficial for the generation of skill, and its retention, in the elderly (Spencer et al., 2007; Fraser et al., 2009; Nemeth et al., 2010; Romano et al., 2010; Wilson et al., 2012), with gains in performance surpassing the gains attained in a single session. Previous studies suggest that the consolidation of motor memories following motor sequence training may be either impaired or less readily mobilized (i.e., selectively engaged) in older adults (Spencer et al., 2007; Harand et al., 2012; Wilson et al., 2012; Korman et al., 2015).

In line with this notion, are the results of studies showing that older adults may need more practice in order to attain a level of mastery in a given task compared to younger trainees (e.g., Rodrigue et al., 2005). However, beyond the quantitative aspects of the training experience (such as the number of sessions or the number of task iterations afforded) other, in a sense, orthogonal, conditions may contribute to the successful learning process in the elderly. Specifically, the affordance of sleep was suggested as a critical modifier of the long-term outcomes of motor skill training, in particular, the ability to express delayed, offline, gains in motor performance in both young (Walker et al., 2003; Korman et al., 2007) and older (e.g., Korman et al., 2015) adults though not in children, i.e., before puberty (e.g., Ashtamker and Karni, 2013).

Both the magnitude of delayed gains and the time-course of the evolution of these gains were shown to be dependent on post-training sleep, either night-time or day-time in young (Walker et al., 2003; Korman et al., 2007; Doyon et al., 2009; Walker and Stickgold, 2010) and older adults (Korman et al., 2015; Mantua et al., 2016). For example, the study by Korman et al. (2015) suggested that while morning training by itself may not constitute a sufficient condition for the subsequent expression of delayed gains in the performance of a newly learned and practiced movement sequence, an interval of day-time sleep afforded shortly after the practice session can make possible the expression of robust delayed gains in the elderly. The authors proposed that the temporal proximity of the practice to a post-training sleep interval is critical, in the elderly; perhaps because opportunities for the interference of subsequent motor experiences in procedural memory consolidation processes (triggered by the practice experience) are reduced. It was conjectured that an increase in the susceptibility to interference by subsequent everyday experience may be one mechanism whereby stricter selectivity about what is or is not prioritized for maintenance in long-term memory is imposed in the elderly, an additional constraint on mnemonic processes (but not learning or the acquisition of within-session, online, gains *per se*) compared to younger adults (Korman et al., 2015).

The results of the current study are in line with the notion that motor training in temporal proximity to a sleep interval may be beneficial to the ability of older adults to retain “how to” motor knowledge. However, the advantage of training at evening hours was not apparent in the overall achievements of the evening trained participants (compared to their peers trained at morning) or the rate of improvement within and between sessions during the multi-session training protocol; the advantage of training at evening was apparent only after more than a month after the termination of practice, i.e., in terms of long-term retention.

Nevertheless, the very fact that in the current study older adults, with a morning orientation in terms of diurnal preferences, managed to acquire a motor skill when trained at evening, at a rate not different from the rate of learning in a morning trained group, raises the need for re-conceptualizing the notion that training at morning is universally the preferable time-of-day for elderly (Roenneberg et al., 2007; Fischer et al., 2017; Hood and Amir, 2017).

Only a few studies have directly addressed the effects of training interventions on the retention of skills, in the elderly. The focus of most studies was on the ability of elderly individual to express within-session, online, gains, or the gains attained by the end of multi-session training programs in comparison to the comparative gains attained by younger adults in similar training regimes (Durkin et al., 1995; Howard et al., 2004; Yan et al., 2010; Ehsani et al., 2015; Korman et al., 2015). A number of studies, however, addressed the retention of practice related gains in task performance in older adults in comparison to younger adults (Shea et al., 2006; Fraser et al., 2009; Nemeth et al., 2010; Wilson et al., 2012). While younger adults were able to retain training-related gains for long periods of time (across time intervals with no additional training), the passage of time (with no additional training afforded) was found to adversely affect the performance gains in older adults (Malone et al., 2016; Griffin et al., 2017). It was concluded that long intervals of no training may lead to forgetting and skill attrition, the loss of gains previously acquired, in the elderly (Malone et al., 2016; Griffin et al., 2017). The current results suggest that perhaps as a default, the motor system of older adults may treat motor “how to” knowledge that is not in continuous use as less prioritized for long-term retention. Given that the long-term maintenance of motor skill in motor cortex is an active and biologically/metabolically “expensive” process and that long-term memory-related plasticity is selectively maintained (Xu et al., 2009; Yu and Zuo, 2011; Yang and Gan, 2012), this may reflect a stricter selectivity in what is to be retained in long-term memory as one ages (see, e.g., Korman et al., 2015).

It is important to stress that the current results were obtained in the socially active and healthy older adult sample, including only participants who did not take frequent day-time naps. However, frequent day-time napping is a common habit among elderly, affecting night-time sleep, cognitive, and physical fitness (Mantua and Spencer, 2017). This may limit the transfer of these findings to a potential clinical setting. Additional factors, such as preventative health medications and some health challenges common to elderly adults that may be related to circadian rhythms (e.g., fatigue, decrements in executive functioning, low physical and social activity) and thus, interfere with the training outcomes, or capacity to engage, should be addressed in the future.

The participants were not asked during the follow-up about their experience with other similar tasks, because finger-tapping tasks are omnipresent, such as in computer and cell phone use. Although the participants were instructed not to practice the experimental task between sessions, the absence of knowledge about the extent of similar experiences between experimental sessions is an additional limitation of the present study. Nevertheless, we note that the participants did not practice the specific sequence of finger movements used in the study task, were not proficient in typing (did not use all fingers for computer work), and had no experimental response box at their disposal (the spatial arrangement of the keys in response box is different from the computer keyboard).

Additional limitation of the current study is that both the training and the assessments were performed at specific time-of-day, only during morning (8–10:30 a.m.) or evening (6:30–9:00 p.m.) hours, for each group. Moreover, the motor task used in this study was a short sequence of fine finger movements; the current results may not generalize to other types of motor training. Responsiveness for physical training and assessment was shown in several recent studies to be dependent on the time of day, suggesting that time-of-day is an important modifier of exercise capacity and associated metabolic pathways (Brito et al., 2019; Ezagouri et al., 2019; Youngstedt et al., 2019).

Thus, our results can serve as a proof-of-concept justifying future studies that will explore conditions or interventions in older adults in which the potential for motor learning, as well as for mastering other types of cognitive tasks, can be harnessed and perhaps facilitated. Specifically, future studies should directly address the possibility, previously raised by Korman et al. (2015), that motor learning and consolidation can be enhanced if elderly participants are restricted in experiencing potentially interfering tasks before the first post-learning sleep interval. The current results also raise the possibility that boost sessions may be beneficial in older adults to better maintain skill in tasks wherein everyday experience is limited or separated by long time intervals. There is evidence underscoring the importance of continuous practice on motor performance in aging; for example, boost sessions were found beneficial for professional pianists to maintain their performance skills, including motor speed, into advanced age (Krampe and Ericsson, 1996) and skilled typists were able to maintain their speed of typing up to 72 years of age, if some practice was afforded every other day (Salthouse, 1996).

We propose that the time-of-day in which practice is afforded, is an important parameter to be considered when it comes to motor skill learning and maintenance in the elderly population; this consideration may also apply in the context of rehabilitation of movement routine (e.g., Korman et al., 2018). On the one hand, morning training may be preferable because it meets the circadian preference of older adults (Roenneberg et al., 2007; Fischer et al., 2017; Hood and Amir, 2017). However, morning training may be less beneficial to the mastering of motor skills, as a long time interval separates the practice experience from the interval of sleep at night; evening training with the proximity to a sleep period may promote better memory consolidation and minimize unspecific interference from everyday motor activity (Korman et al., 2015). These considerations can be taken into account by practitioners in structuring home/rehabilitation programs for older adults, making the scheduling of the intervention sessions to morning hours less restrictive. The time-of-day of practice, specifically in the rehabilitation of function in elderly individuals who are less well-entrained to the environmental cues of the diurnal rhythms (Camargo-Sanchez et al., 2015; Duffy et al., 2015; Mattis and Sehgal, 2016; Cornelissen and Otsuka, 2017; Logan and McClung, 2019) may need to be addressed as a critical factor in the studies of long-term effects of intervention protocols.

## Data Availability Statement

All datasets generated for this study are included in the article/supplementary material.

## Ethics Statement

The studies involving human participants were reviewed and approved by the University of Haifa, Human Experimentation Ethics Committee of the Natural Sciences Faculty. The patients/participants provided their written informed consent to participate in this study.

## Author Contributions

All authors read and approved the final version of the manuscript. CG, AK, and MK conceived and designed the experiments. CG collected the data. CG and EG analyzed the raw data. CG, RM-H, AK, and MK made the statistical analysis and interpretation of the data. CG, EG, AK, and MK wrote the article.

## Conflict of Interest

The authors declare that the research was conducted in the absence of any commercial or financial relationships that could be construed as a potential conflict of interest.
